# Elevated homocysteine and depression outcomes in patients with comorbid medical conditions in rural primary care

**Published:** 2022-07-22

**Authors:** Krishnamachari Srinivasan, Luke Joshua Salazar, Elsa Heylen, Maria L. Ekstrand

**Affiliations:** 1 Department of Psychiatry, St. John’s Medical College; 2 Division of Mental Health and Neurosciences, St. John’s Research Institute, Bengaluru, Karnataka, India; 3 Department of Medicine, Division of Prevention Sciences, University of California, San Francisco, California, USA

**Keywords:** Depression, homocysteine, primary health care

## Abstract

We examined the association of elevated concentration of total homocysteine (tHcy) with the severity of depression in patients diagnosed with depression and comorbid chronic medical conditions in rural primary care settings in Karnataka. Participants were included from the control arm of a cluster-randomized controlled trial designed to evaluate the effects of using a collaborative care model to integrate screening and treatment of primary health center patients. tHcy was assayed at baseline, and depression severity scores were assessed using the Patient Health Questionnaire (PHQ-9) 6 months later. There was no difference in the mean PHQ-9 score between those with (mean PHQ = 7.4) and without (mean PHQ = 7.6) elevated tHcy levels (*P* = 0.67).

## Introduction

An elevated concentration of total homocysteine (tHcy) has been shown to be associated with an increased risk for several neuropsychiatric conditions including depression.^[1]^ A recent meta-analysis of 46 observational studies that included population-based and clinic-based studies found that subjects diagnosed with depression had a significantly elevated concentration of homocysteine compared to healthy controls (weighted mean difference = 2.53 μmol/L, 95% confidence interval [CI]: 1.77, 3.30), and individuals with elevated concentration of homocysteine had a significantly higher risk of developing depression (pooled risk = 1.34, 95% CI: 1.19, 1.52).^[2]^

A few community-based studies have reported an association between tHcy concentrations and severity and course of depressive symptoms.^[3–6]^ A study from Australia noted that individuals in the lowest quartile of tHcy reported fewer depressive symptoms adjusted for several confounders,^[3]^ but others failed to find a significant association between serum tHcy concentrations and depressive symptoms,^[4]^ while another study reported an association only among older subjects (≥50 years).^[5]^ A longitudinal study among men and women aged 65 years or older did not find any associations between baseline tHcy concentrations and either change in depression score over 16 years or incident depression.^[6]^

To our knowledge, elevated concentration of tHcy as a predictor of the severity of depression in patients diagnosed with depression and comorbid chronic medical conditions has not been studied. The aim of the present study was to address this gap in the literature.

## Methods

### Setting and participants

The present study was a part of a cluster-randomized controlled trial (cRCT) designed to evaluate the effects of using a collaborative care model to integrate screening and treatment of primary health center (PHC) patients with depression who were also diagnosed with chronic medical conditions, in rural Karnataka.^[7]^ The study was conducted in 49 PHCs in the rural Ramnagara district of Karnataka state in southern India. PHCs were randomly selected and were randomized 1:1 into the collaborative care and “enhanced” standard care arms. Intervention (collaborative care) arm PHC staff were trained in mental health diagnosis, treatment, and the collaborative care model. All staff in the PHCs that were randomized to the control (enhanced standard care) arm received a full day of basic training in established clinical protocols for the management of depression. The present analysis included 205 participants from the “enhanced” standard care arm with Patient Health Questionnaire (PHQ-9) scores of at least 10 and with 6-month follow-up data available. Participants were adults (≥30 years) with depression and comorbid medical conditions that included either hypertension, diabetes, or ischemic heart disease.

### Measures

We collected demographic details of the participants including age, gender, marital status, educational status, and household income. The Generalized Anxiety Disorder Scale and the PHQ-9 Depression Scale were used to assess the severity of anxiety and depression, respectively, at baseline and 6-month follow-up.^[8,9]^ We had collected 10 ml of venous blood at baseline as part of the parent study to perform laboratory diagnostic tests and blood was stored at −80°C.

### Biochemistry assessments

#### Homocysteine (μmol/L)

tHcy was analyzed by enzymatic method on Cobas Integra chemistry autoanalyzer with dedicated reagent from Roche Diagnostics (Switzerland). Two levels of tHcy control were used. Within-day imprecision was <5% and between-day imprecision was 4.02% and 4.0% for Level 1 (12.85 ± 0.52) and Level 2 (40.03 ± 1.60), respectively.

#### Folate (ng/mL) and Vitamin B12 (pg/mL)

Folate and Vitamin B12 were analyzed by electrochemiluminescence on Cobas e411 immunoassay analyzer with dedicated reagents from Roche Diagnostics (Switzerland). Within-day imprecision for folate was <10% and between-day imprecision was 10.84%, 14.23%, and 15.92% for Level 1 (mean 2.65 ± 0.29), Level 2 (mean 7.55 ± 1.07), and Level 3 (mean: 15.85 ± 2.52), respectively. For Vitamin B12, within-day imprecision was 10% and between-day imprecision was 5.84%, 5.30%, and 4.90% for Level 1 (mean: 391.73 ± 22.89), Level 2 (mean: 548.51 ± 29.07), and Level 3 (mean: 701.51 ± 34.35), respectively.

### Statistical analysis

Descriptive statistics used were mean with standard deviation for continuous variables, and frequencies and percentages for categorical variables. Independent sample’s *t*-test and linear regression were used to examine the association between elevated baseline tHcy concentrations and PHQ-9 scores at 6 months, overall, and with age as a moderator. All analyses were performed using IBM SPSS Statistics for Windows, Version 25.0. (Armonk, NY: IBM Corp).

### Ethical considerations

Ethics approval was obtained from the Institutional Ethical Review Board at St. John’s Medical College and Hospital, and the Committee on Human Research, University of California, San Francisco. All participants provided written informed consent.

## Results

The baseline characteristics of the study participants are shown in Table 1.

There was no difference in the mean PHQ-9 score between those with (mean: PHQ = 7.4) and without (mean: PHQ = 7.6) elevated tHcy levels (*P* = 0.67). There was no significant interaction between age and elevated tHcy on depression severity (*P* = 0.44). Among those aged ≥65 years with elevated tHcy (*n* = 38), PHQ scores tended to be higher compared to those with normal tHcy concentrations (*n* = 32) (8.2 ± 3.2 vs. 7.8 ± 2.9, *P* = 0.65).

## Discussion

In our sample of patients with clinical depression, elevated tHcy did not predict the severity of depression 6 months later. Similar findings were reported in another study, in which no significant associations were found between tHcy concentrations and change in depression scores over 16 years, as captured by the Center for Epidemiologic Studies Depression Scale^[6]^ In addition, a meta-analysis reported that tools used to measure depression severity can be a source of heterogeneity, with studies that used PHQ-9 as a measure of depressive symptoms failing to find associations between tHcy concentrations and depression.^[2]^

Although, in the present study, age was not found to have a significant influence on the relationship between elevated tHcy and the severity of depression, others have reported a significant and positive association between tHcy concentrations and elevated depressive symptoms only among the elderly.^[5]^

While our study is limited by the relatively small sample size, it is worthwhile exploring the association between elevated tHcy concentrations and severity of depression, particularly among elderly subjects in future studies.

## Figures and Tables

**Table 1:** Description of the sample at baseline (*n*=205)

	*n* (%)
Female	159 (77.6)
Age (years)	
30–44	21 (10.2)
45–54	38 (18.5)
55–64	76 (37.1)
≥65	70 (34.1)
Married	143 (69.8)
Hindu religion	196 (95.6)
Education level (*n*=204)	
No formal education	113 (55.4)
Primary	66 (32.4)
Secondary or higher	25 (12.3)
Household income ≤5000 INR	143 (69.8)
Elevated tHcy^*^	105 (51.2)
Folate deficiency^†^ (*n*=200)	133 (66.5)
Vitamin B12 deficiency^‡^	18 (8.8)
GAD-7 anxiety score (0–21)^§^	10.0 (3.7)
PHQ-9 depression score (0–27)^§^	13.4 (3.2)

* Elevated tHcy: 18–65 years: ≥15 mmol/l, >65 years: ≥20 mmol/l

† Folate deficiency: male: <4.5 ng/ml, Female: <4.8 ng/ml

‡ Vitamin B12 deficiency: <197 pg/ml

§ Mean±SD. tHcy – Total homocysteine, GAD-7 – Generalized Anxiety Disorder Scale-7, PHQ-9 – Patient Health Questionnaire, SD – Standard deviation
